# Antimicrobial stewardship during COVID-19: An analysis of culture negative patients receiving extended antimicrobial agents

**DOI:** 10.1017/ash.2023.275

**Published:** 2023-09-29

**Authors:** Swetha Srialluri, Curtis Collins, Holly Murphy

## Abstract

**Background:** COVID-19 is associated with symptoms, clinical findings, and laboratory abnormalities that raise concern for secondary infections. Excess antimicrobial use despite low rates of secondary infections has been reported and presents a continuing challenge for antimicrobial stewardship programs (ASPs), particularly during COVID-19 surges. The objective of this study was to analyze the appropriateness of antimicrobial use in patients with extended antimicrobial therapy during 2 distinct COVID-19 hospital surges. **Methods:** We conducted an observational, retrospective, cohort study of COVID-19 patients admitted to our 548-bed community teaching hospital between November and December 2021 (ie, the SARS-CoV-2 delta-variant predominant phase) and January–February 2022 (ie, the SARS-CoV-2 omicron-variant predominant phase) and who received antibiotics for >4 days without positive cultures. Demographic and clinical data were obtained from the institutional data warehouse. Infectious diseases–trained researchers evaluated the appropriateness of antimicrobials based on diagnostic and clinical reporting and institutional antimicrobial stewardship guidelines. Patients were considered to have probable secondary bacterial infection if they had 2 of the following symptoms: fever, unexplained leukocytosis, worsening secretions, or hypoxia and/or imaging. The outcomes of interest included confirmed infections and excess antimicrobial days. Categorical and continuous variables were analyzed using χ^2^ tests, Fisher exact tests, and Mann-Whitney *U* tests, respectively. Statistical significance was defined as *P* ≤ .05. **Results:** In total, 87 patients were included in the study. Moreover, 56 patients were identified in the SARS-CoV-2 delta-variant predominant phase and 31 patients were identified in the SARS-CoV-2 omicron-variant predominant phase. The groups were similar, with higher vaccination rates in the SARS-CoV-2 omicron-variant predominant group (37.5% vs 64.5%; *P* = .016). Patients in the SARS-CoV-2 omicron-variant predominant group required less mechanical ventilation (39.3% vs 16.1%; *P* = .025). There were no significant differences in infectious diseases consultation, immunomodulator or remdesivir use, antimicrobials classes prescribed, or antimicrobial days of therapy or duration between cohorts. There were no significant differences in length of stay, 30-day mortality, or 30-day readmissions. Infections were confirmed in 78.6% in the delta-variant group versus 83.9% in the omicron-variant group (*P* = .55). Pneumonias accounted for 60.7% in the delta-variant group and 40.9%, in the omicron-variant group. Excess antibiotic use occurred in 14.3% of patients in the delta-variant group and in 3.1% of patients in the omicron-variant group (*P* = .149). There was no significant difference in the duration of inappropriate antimicrobial use between groups in patients without infections: 5 days in the delta-variant group versus 5 days in the omicron-variant group (*P* = .24). **Conclusions:** Results demonstrated that most antimicrobial use was appropriate in a challenging patient population lacking positive cultures to guide therapy. Inappropriate antimicrobial utilization occurred demonstrating continued opportunities for our institutional ASP.

**Disclosures:** None

